# Analysis of Intestinal and Nasopharyngeal Microbiota of Children with Meningococcemia in Pediatric Intensive Care Unit: INMACS-PICU Study

**DOI:** 10.3390/diagnostics13121984

**Published:** 2023-06-06

**Authors:** Gurkan Bozan, Vicente Pérez-Brocal, Kaan Aslan, Eylem Kiral, Esra Sevketoglu, Mutlu Uysal Yazici, Ebru Azapagasi, Tanil Kendirli, Serhat Emeksiz, Oguz Dursun, Dincer Yildizdas, Ayse Berna Anil, Nihal Akcay, Hasan Serdar Kihtir, Merve Havan, Nazan Ulgen Tekerek, Faruk Ekinci, Omer Kilic, Andres Moya, Ener Cagri Dinleyici

**Affiliations:** 1 Pediatric Intensive Care Unit, Faculty of Medicine, Eskisehir Osmangazi University, Eskisehir 26040, Turkey; 2 Area of Genomics and Health, Foundation for the Promotion of Sanitary and Biomedical Research of Valencia Region (FISABIO-Public Health), 46020 Valencia, Spain; 3 Biomedical Research Networking Center for Epidemiology and Public Health (CIBEResp), 28029 Madrid, Spain; 4 Department of Pediatrics, Faculty of Medicine, Eskisehir Osmangazi University, Eskisehir 26040, Turkey; 5 Pediatric Intensive Care Unit, Bakirkoy Dr. Sadi Konuk Training and Research Hospital, University of Health Sciences, Istanbul 34147, Turkey; 6 Pediatric Intensive Care Unit, Faculty of Medicine, Gazi University, Ankara 06500, Turkey; 7 Pediatric Intensive Care Unit, Faculty of Medicine, Ankara University, Ankara 06590, Turkey; 8 Pediatric Intensive Care Unit, Ankara City Hospital, Ankara 06800, Turkey; 9 Pediatric Intensive Care Unit, Faculty of Medicine, Akdeniz University, Antalya 07070, Turkey; 10 Pediatric Intensive Care Unit, Faculty of Medicine, Cukurova University, Adana 01790, Turkey; 11 Pediatric Intensive Care Unit, Faculty of Medicine, Izmir Katip Celebi University, Izmir 35620, Turkey; 12 Department of Pediatric Critical Care, Antalya Training and Research Hospital, University of Health Sciences, Antalya 07100, Turkey; 13 Division of Pediatric Infectious Diseases, Faculty of Medicine, Eskisehir Osmangazi University, Eskisehir 26040, Turkey; 14 Institute for Integrative Systems Biology (I2SysBio), University of Valencia and Spanish National Research Council (CSIC), 46010 Valencia, Spain

**Keywords:** gut microbiota, nasopharyngeal microbiota, sepsis, meningococcal, Neisseria meningitidis, children

## Abstract

Microbiota composition might play a role in the pathophysiology and course of sepsis, and understanding its dynamics is of clinical interest. Invasive meningococcal disease (IMD) is an important cause of community-acquired serious infection, and there is no information regarding microbiota composition in children with meningococcemia. In this study, we aimed to evaluate the intestinal and nasopharyngeal microbiota composition of children with IMD. **Materials and Methods:** In this prospective, multi-center study, 10 children with meningococcemia and 10 age-matched healthy controls were included. Nasopharyngeal and fecal samples were obtained at admission to the intensive care unit and on the tenth day of their hospital stay. The V3 and V4 regions of the 16S rRNA gene were amplified following the 16S Metagenomic Sequencing Library Preparation. **Results:** Regarding the alpha diversity on the day of admission and on the tenth day at the PICU, the Shannon index was significantly lower in the IMD group compared to the control group (*p* = 0.002 at admission and *p* = 0.001, on the tenth day of PICU). A statistical difference in the stool samples was found between the IMD group at Day 0 vs. the controls in the results of the Bray–Curtis and Jaccard analyses (*p* = 0.005 and *p* = 0.001, respectively). There were differences in the intestinal microbiota composition between the children with IMD at admission and Day 10 and the healthy controls. Regarding the nasopharyngeal microbiota analysis, in the children with IMD at admission, at the genus level, *Neisseria* was significantly more abundant compared to the healthy children (*p* < 0.001). In the children with IMD at Day 10, genera *Moraxella* and *Neisseria* were decreased compared to the healthy children. In the children with IMD on Day 0, for paired samples, *Moraxella*, *Neisseria*, and *Haemophilus* were significantly more abundant compared to the children with IMD at Day 10. In the children with IMD at Day 10, the *Moraxella* and *Neisseria* genera were decreased, and 20 different genera were more abundant compared to Day 0. **Conclusions:** We first found alterations in the intestinal and nasopharyngeal microbiota composition in the children with IMD. The infection itself or the other care interventions also caused changes to the microbiota composition during the follow-up period. Understanding the interaction of microbiota with pathogens, e.g., *N. meningitidis*, could give us the opportunity to understand the disease’s dynamics.

## 1. Introduction

Due to its widespread distribution, epidemic potential, fulminant clinical manifestations, association with high case-fatality rates, and long-term sequelae among survivors, invasive meningococcal disease (IMD), caused by *Neisseria meningitidis*, is a significant cause of sepsis and septic shock in children [1,2]. Based on the immunochemistry and genetics of the *Neisseria meningitidis* capsular polysaccharides, twelve serogroups have been identified, with six (A, B, C, W, X, and Y) accounting for the majority of all cases of IMD around the world [1,2]. The geographical distribution and epidemic potential of Nm strains differ. In Turkey, serogroup B and serogroup W are the predominant consecutive serogroups in IMD, and serogroups A, Y, and X have also been reported as causes [3]. Serogroups W, B, and X are also important causes of nasopharyngeal meningococcal carriage in Turkey [4,5].

The microbiota composition, especially that of the gut microbiota, has been shown to play a role in the pathogenesis and prognosis of many diseases, including infectious diseases [6,7,8]. Increasing evidence in clinical studies in recent years points to the microbiota as an important player in the pathophysiology of sepsis [7,9,10,11,12,13]. Considering the protective effects of a healthy intestinal microbiota on colonization resistance and systemic immunity, it has been suggested that the disruption of the integrity of the microbiota potentially increases susceptibility to sepsis. The incompatibility and deterioration of these components may explain the pathogenesis of sepsis and organ dysfunction. However, the specific mechanisms that cause the deterioration of intestinal homeostasis that progresses to sepsis remain unclear [9,14]. 

However, the mechanism of the gut microbiome’s function in sepsis remains unknown. IMD is also an important example for the better understanding of serious infection and sepsis, which presents with shock within several hours, requiring intensive care. Microbiota composition might play a role in the pathogenesis and prognosis of IMD; however, there is no study examining the microbiota changes in either stool or nasopharyngeal microbiota in patients with IMD. The aim of this study was to determine the changes and compositional differences in both intestinal and nasopharyngeal microbiota in children with meningococcal septicemia, requiring pediatric intensive care stays.

## 2. Materials and Methods

### 2.1. Study Design

The INMACS-PICU study was a prospective, multicenter, observational study, which aimed to evaluate microbiota composition in children with sepsis and septic shock between August 2018 and August 2020. Nine tertiary care centers from seven cities in Turkey agreed to participate in this study. Children between the ages of 3 and 18 years who were admitted to the pediatric intensive care unit with a clinical suspicion of sepsis or septic shock were evaluated. Children with a diagnosis of sepsis due to meningococcemia who underwent culture or molecular tests in the first 48 h of their admission to the pediatric intensive care unit (PICU) were included in this part of the study. Patients younger than three years of age, all patients who required medication or had a special diet for specific needs, especially those with hematological oncological malignancies (leukemia, lymphoma, congenital heart disease, celiac, inflammatory bowel disease), patients with a BMI above the 95th percentile for their age, patients with malnutrition, and those who had used probiotics or antibiotics in the 8 weeks before the application date due to dysbiosis were excluded from the study. Ten healthy children over 3 years of age, who did not have a chronic disease, were born by normal spontaneous vaginal delivery, had received only breast milk for their first 6 months, and had been given antibiotics or probiotics in the previous 8 weeks were included in the study as the control group. Stool and nasopharyngeal swab samples were taken from the subjects who met the inclusion criteria, considering the inclusion day as Day 0. The meningococcemia treatment protocols were quite similar among the cities, including fluid and antibiotic treatment. Ceftriaxone is the first-line antibiotic treatment in all centers for patients who have suspected IMD.

This study was planned in accordance with the Helsinki Declaration and patient rights regulations and ethical committees, and ethics committee approval was obtained from the Eskisehir Osmangazi University Faculty of Medicine (27 February 2018, decision no. 54). Written informed consent was obtained from the parents of all children participating in the study. This study was supported by the Eskisehir Osmangazi University Scientific Research Grant (2018/11046).

### 2.2. Sample Collection

From the study group, stool and nasopharyngeal swab samples were taken on the first day of admission and, for survivors, on the tenth day of their hospital stay. Stool and nasopharyngeal swab samples were taken once from the control group. The stool samples obtained from the participants were taken in 50 cc empty Falcon tubes, with at least 5 mL, and the Falcon tubes were stored upright at −80 °C without any treatment. Nasopharyngeal samples were obtained from the participants in a previously prepared 1.5 mL solution. All the samples were delivered to the laboratory where DNA analysis was conducted in accordance with cold chain requirements (storing at −80 °C) every three months. 

### 2.3. DNA Extraction

The fecal samples were processed using QuickGene (Kurabo, Osaka, Japan), which was used to extract DNA from the stool samples. First, 25 mg of each stool sample was transferred to a homogenization tube with 250 µL of tissue lysis solution. To homogenize the solution, 15 mg of 0.1 mm glass beads or 10 1.0 mmø zirconia beads were added to the tube and then homogenized for 2 × 120 s at 5000 rpm. After the sample was homogenized, 25 µL of proteinase K solution was added and incubated at 56 °C for 60 min. The tube was then centrifuged at 15,000× *g* for 10 min at room temperature. After centrifugation, 200 µL of supernatant was transferred to a 1.5 mL microtube. Then, 180 µL of cell lysis solution was added and vortexed for 15 s. The microtube was left to incubate at 70 °C for 10 min. In the next step, 240 µL of 99% cold ethanol was added and vortexed for 15 s. The entire contents of the microtube were transferred to a QuickGene (Kurabo, Osaka, Japan) filtered cassette, where washes and elutions were performed following the instrument’s protocol. Three washes were performed using 750 μL of wash buffer solution. Based on the results of the extraction process, bacterial 16S ribosomal RNA (rRNA) gene target sequencing was performed using the materials obtained in the study.

For the nasopharyngeal samples, during extraction, the samples were thawed and diluted in 9 mL of phosphate-buffered saline (PBS). The samples suspended in PBS were then centrifuged at 400× *g* for 10 min at room temperature. At the end of centrifugation, the supernatants were discarded, and the study was continued with pellets. Then, genomic DNA was obtained using a pellet Kurabo QuickGene DNA tissue kit S. The pellets were applied to 250 µL of MDT and 25 µL of proteinase K solution and incubated at 56 °C for 60 min. After adding 180 µL of LDT solution, the tubes were mixed for 15 s and incubated at 70 °C for 10 min. A total of 240 µL of 99% cold ethanol was added to the tubes and mixed for 15 s. Immediately afterwards, the entire volume in the microcentrifuge tube was transferred to QuickGene columns and mixed with 750 µL of wash buffer solution three times. Afterwards, 200 µL of CDT solution was added to QuickGene columns, and 50–60 ng of genomic DNA was collected in a new sterile 1.5 mL microcentrifuge tube.

### 2.4. Library Preparation, Sequence Analysis, and Bioinformatics

Following the 16S Metagenomic Sequencing Library Preparation Illumina technique (Illumina, San Diego, CA, USA), the V3 and V4 sections of the 16S rRNA gene were amplified. Parallel to the samples, the extraction controls were amplified and sequenced.

A 16S Amplicon PCR Forward Primer (5′-TCGTCGGCAGCGTCAGATGTGTATAAGAGACAGCCTACGGGNGGCWGCAG-3′) and a 16S Amplicon PCR Reverse Primer (5′-GTCTCGTGGGCTCGGAGATGTGTATAAGAGACAGGACTACHVGGGTATCTAATCC-3′) were the primers used to target this region.

The following PCR conditions were employed for the amplification of a total of 12.5 ng of genomic DNA per sample: the initial denaturation for 5 min at 94 °C, followed by 25 cycles of denaturation (30 s at 94 °C), annealing (30 s at 52 °C), and elongation (1 min at 72 °C). The products were seen in 1.4% agarose gels after amplification and quantified with a Qubit^®^ 3.0 Fluorometer (Thermo Fisher Scientific, Carlsbad, CA, USA). After adding dual indices to both ends of the PCR products, the Nextera XT Index Kit (Illumina) was used to complete the multiplexing phase. According to the manufacturer’s instructions, the samples were combined into equimolar quantities and sequenced in the Sequencing Service facilities of FISABIO using a 2 × 300 bp paired-end run, utilizing the MiSeq^®^ Reagent kit v3 (Illumina) on a MiSeq Sequencer (Illumina). Internal controls were used during the extraction, followed by PCR (with a negative control) and sequencing processes. We employed an internal control for 16S during sequencing.

The demultiplexed forward and reverse fastq files containing reads in matched order, free of primer, adapter, and linker sequences were the input files for the DADA2 pipeline [15]. DADA2 was used to analyze the quality profiles and to filter and trim the Ns, expected errors, and low-quality tails. After learning the error rates with the DADA2 algorithm, a dereplication step was used to reduce the computation time by collapsing redundant reads (although they were counted). Next, true sequence variants were inferred, paired reads were merged by aligning the denoised forward and reverse reads with a minimum overlapping set in 15 identical bases, and the amplicon sequence variant table was constructed. Chimeric sequences were identified and removed. Before taxonomic assignment, bowtie2-2.3.4.2 was used with end-to-end and highly sensitive parameters to map the reads against the human genome (GRCh38.p11, reference human genome, December 2013) [16]. We then implemented taxonomic assignment of the unaligned reads by the naïve Bayesian classifier method using the Silva reference database and extending the assignment to species level when possible. Counts were obtained for amplicon sequence variants (ASVs) and collapsed to the species level. After extracting the counts for each taxon in each sample from the resulting files, a special contingency table was created. Using the QIIME pipeline version 1.9.0 for composition and abundance analyses as well as for ecological diversity [17], the contingency table was translated into the Biom format. Of 9500 random reads per sample, with replacement, 1000 rarefactions were performed for diversity within samples, and the alpha diversity was estimated using the Shannon diversity index. Using the free R 3.1.0 statistical software packages [18], differential abundance analyses of the ASVs, species, and genera were carried out using ANCOM-BC with false discovery rate (FDR) correction [19] for paired comparisons between the samples from subjects at Day 0 and Day 10, and for unpaired comparison between the children with meningococcemia and the controls. Signal filtering of the variables was applied to normalized ANCOM-BC data, and adjusted *p*-values were recalculated. Finally, taxa showing adjusted *p*-values < 0.05 were plotted together with their log2 fold change as volcano plots for each pair of comparisons, at the genus level, using the VolcaNoseR web app [20]. In addition, R 3.1.0 software was used to calculate the Bray–Curtis distance for the beta diversity, and principal coordinates analyses (PCoA) were performed to visualize the differences among the groups, including Adonis nonparametric analysis of variance to determine the statistical significance of sample groupings within the data.

## 3. Results

We screened 32 children with sepsis/septic shock requiring pediatric intensive care. A total of 22 children were excluded because their sepsis was caused by other microorganisms. Ten children (five girls and five boys, aged 3 to 14 years) with meningococcemia and ten healthy controls (five girls and five boys, aged 3 to 15 years) who met the criteria for inclusion in the study were enrolled. A flow chart is provided based on Strengthening the Organization and Reporting of Microbiome Studies (STORMS) [21] (Supplementary Figure S1). There was no statistical difference between the patient and control groups in terms of age (*p* > 0.05). All children in the meningococcemia group had clinical and laboratory findings of sepsis and required a pediatric intensive care unit stay. All had a fever and non-blanching rash at the admission. The healthy children were required to have been vaginally delivered and breast-fed during their first 6 months of life (according to the inclusion criteria). In the meningococcemia group, seven children were born via C-section, and four children were exclusively breastfed during the first 6 months of life. Regarding the serogroup distribution of the ten children with meningococcemia, eight children had *N. meningitidis* due to serogroup B, one child due to serogroup W, and the serogroup of one child was non-groupable. None of the children had increased risk conditions for IMD, such as asplenia, complement deficiencies, HIV, etc., and had been previously healthy. 

### 3.1. Intestinal Microbiota Analysis

Regarding the alpha diversity on the day of admission and on the tenth day at the PICU, the Shannon index (a measure of richness and uniformity that considers the entropy) was significantly lower in the IMD group compared to the control group (*p* = 0.002 and *p* = 0.001, respectively). There was no difference in the IMD patients between admission and on the tenth day of their stay. No significance was observed for the Chao1 index between the children with IMD at admission and the healthy controls (*p* > 0.05). However, there was a difference in the Chao1 index of children with IMD on the tenth day in the PICU and the healthy controls (*p* = 0.001) (Table 1).

A statistical difference in the stool samples was found between the groups in the results of the Bray–Curtis (meningococcemia group vs. control *p* = 0.005, Day 0) and Jaccard analyses (meningococcemia group vs. control *p* = 0.001, Day 0) for the beta diversity between the children with IMD at admission and the healthy controls (*p* < 0.05 for both). A statistical difference in the beta diversity of the nasopharyngeal samples was found between the groups in the results of the Bray–Curtis (meningococcemia group, Day 0 vs. Day 10; *p* = 0.006) and Jaccard analyses (meningococcemia group, Day 0 vs. Day 10; *p* = 0.007 and meningococcemia group vs. control *p* = 0.032, Day 0). 

**Figure 1:**
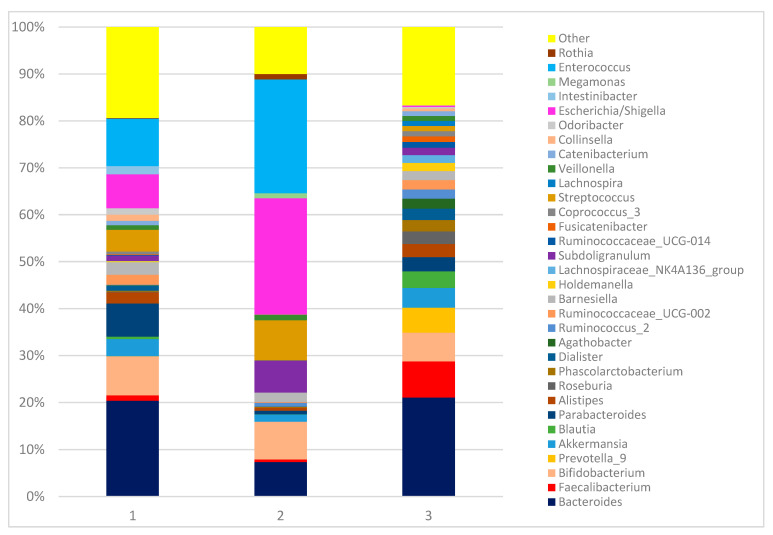
Intestinal microbiota compositions of children with IMD on Day 0 (1) and on Day 10 (2), and healthy controls (3) at genus level. Relative abundance analysis of the bacterial community at genus level (relative abundance > 1%; bacteria with relative abundances < 1% were pooled in the “Other” category and sorted by total concentration).

The intestinal microbiota compositions of the children with IMD upon admission (Day 0) (1) and on Day 10 (2), and of the healthy controls (3) at the genus level are shown in Figure 1. 

**Figure 2:**
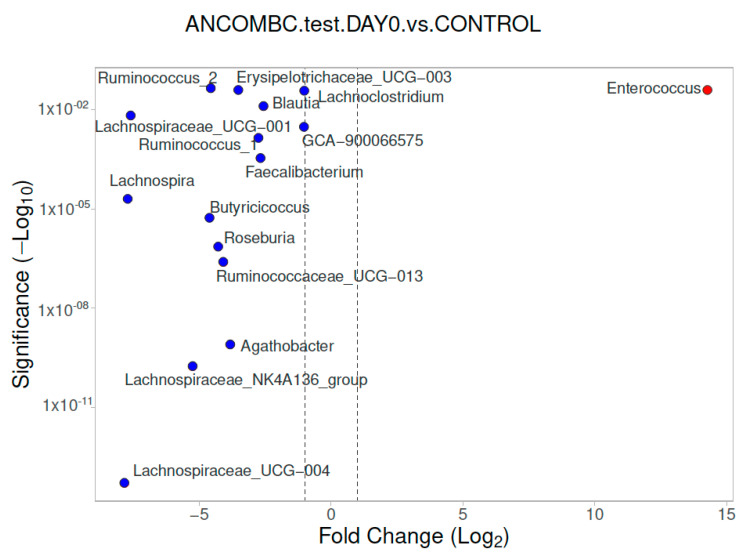
Differential abundance of stool analysis at genus level, calculated using ANCOM-BC, between children with IMD at Day 0 and healthy controls, with volcano plots. Significant genera are plotted according to their log_2_ fold change and adjusted *p*-values. Red dots: IMD on Day 0; blue dots: healthy children.

**Figure 3:**
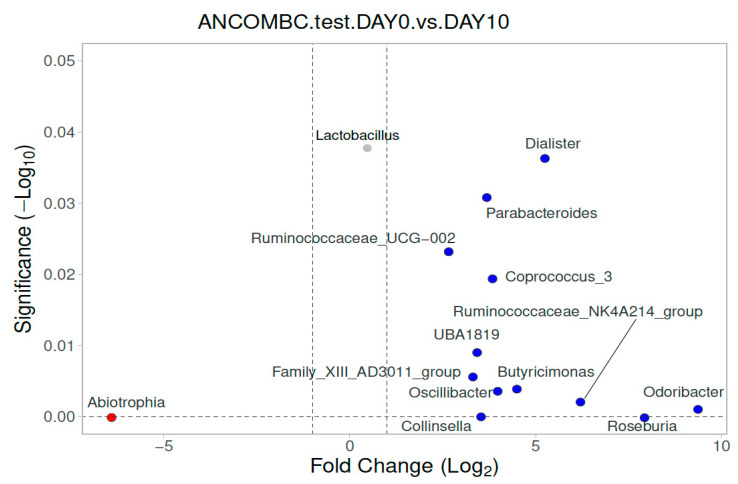
Differential abundance of stool analysis at genus level, calculated using ANCOM-BC, between children with IMD at Day 0 and Day 10 (paired samples), with volcano plots. Significant genera are plotted according to their log_2_ fold change and adjusted *p*-values. Red dots: IMD at Day 0; blue dots: IMD at Day 10. Grey dots mean that their log_2_ fold change was lower than 1, low in magnitude, but significant.

**Figure 4:**
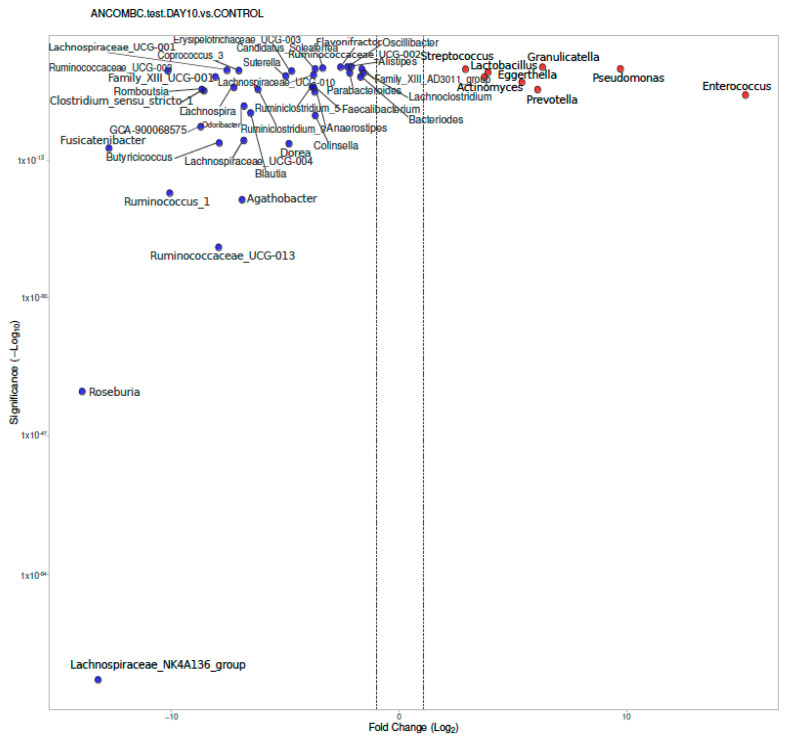
Differential abundance of genera from stool samples, calculated using ANCOM-BC, between children with IMD at Day 10 and healthy controls, with volcano plot. Significant genera are plotted according to their log_2_ fold change and adjusted *p*-values. Red dots: IMD at Day 0; blue dots: healthy children.

Differential abundance analysis of the genera was carried out using ANCOM-BC for paired comparisons between the samples from subjects at day 0 and day 10, and unpaired comparisons between the children with meningococcemia and the controls. There was a difference between the children with IMD at admission and the healthy controls. The volcano plot graphics show the bacterial taxa that were significantly different in abundance between the study groups (Figure 2, Figure 3 and Figure 4). At the genus level, in the children with IMD at admission, *Enterococcus* were found to be dominant (*p* < 0.05). In the healthy controls, *Lachnospiraceae UCG-004, Lachnospiraceae NK4A136 group, Agathobacter, Ruminococcaceae UCG-013, Roseburia, Butyricicoccus, Lachnospira, Faecalibacterium, Ruminococcus_1, Lachnospiraceae GCA-9000066575, Blautia, Lachnoclostridium, Erysipelotrichaceae_UCG-003, Anerostipes*, and *Ruminococcus_1* were found to be significantly dominant, compared to the children with IMD at admission (Figure 2).

For the intestinal microbiota composition, there was also a difference in the paired samples between the children with IMD at admission (Day 0) and at Day 10 (Figure 3). In the children with IMD at admission, *Roseburia, Collinsella, Odoribacter, Oscillibacter, Butryicimonas, Ruminococcaceae UBA1819, Coprococcus 3, Ruminococcaceae UCG-002, Parabacteroides, Dialister,* and *Lactobacillus* were found to be dominant compared to Day 10. On the other hand, at Day 10, *Abiotrophia* was found to be dominant compared to the baseline.

There was a difference between the children with IMD at Day 10 and the healthy controls. At the genus level, in the children with IMD at Day 10, *Enterococcus, Pseudomonas, Granulicatella, Prevotella, Eggerthella, Actinomyces, Lactobacillus,* and *Streptococcus* were found to be significantly dominant (Figure 4). In the healthy controls, *Lachnospiraceae NK4A136 group, Roseburia, Ruminococcaceae UCG-013, Agathobacter, Ruminococcus_1,* and more than 20 different genera (shown in Figure 4) were found to be significantly dominant compared to the children with IMD at Day 10.

### 3.2. Nasopharyngeal Microbiota Analysis

For the alpha diversity, at Day 0 and Day 10 in the PICU, the Shannon index (a measure of richness and uniformity that considers entropy) of the IMD group was similar to that of the control group (*p* > 0.05). The Shannon index was lower in the children with IMD at Day 10 compared to the baseline (*p* = 0.001). The Chao1 index of the children with IMD at the baseline was significantly lower than the children with IMD at Day 10 (*p* = 0.002, Table 1).

A statistical difference in the nasopharyngeal samples was found for the beta diversity in the results of the Bray–Curtis (*p* < 0.05) and Jaccard analyses (*p* < 0.05) between the children with IMD at admission and Day 10. There was also a difference between the children with IMD at admission and the healthy controls (*p* < 0.05).

**Figure 5:**
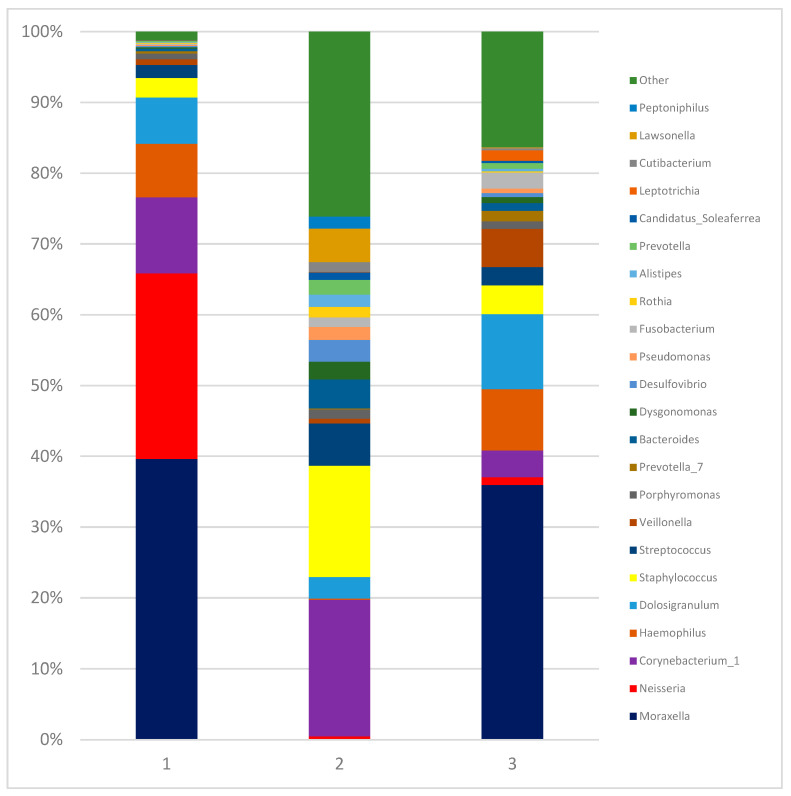
Nasopharyngeal microbiota composition of children with IMD at admission (1), at Day 10 (2), and healthy controls (3) at genus level. Relative abundance analysis of the bacterial community at genus (relative abundance > 1%; bacteria with relative abundances < 1% were pooled in the “Other” category and sorted by total concentration).

The nasopharyngeal microbiota compositions of the children with IMD at Day 0 (1) and Day 10 (2) and the healthy controls (3) at the genus level are shown in Figure 5. 

**Figure 6:**
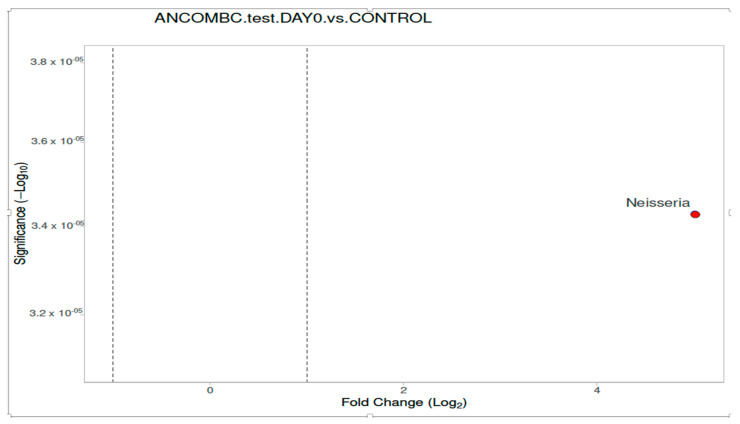
Differential abundance of nasopharyngeal microbiota analysis at genus level, calculated using ANCOM-BC, between children with IMD at Day 0 and healthy controls, with volcano plots. Significant genera are plotted according to their log_2_ fold change and adjusted *p*-value. Red dots: IMD at Day 0.

**Figure 7:**
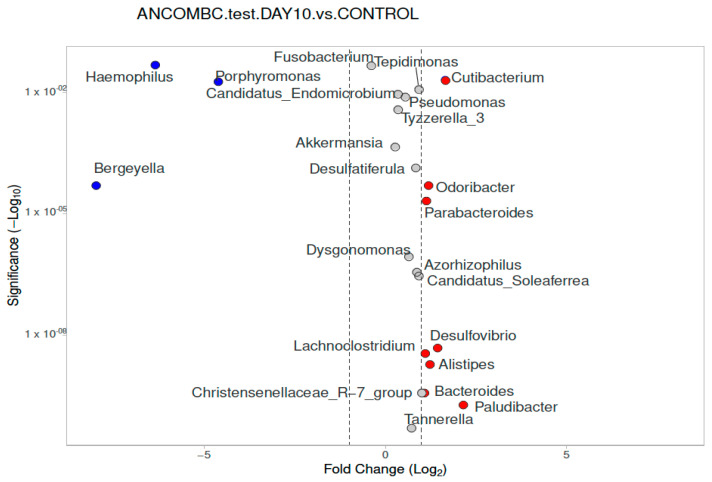
Differential abundance of nasopharyngeal microbiota analysis at genus level, calculated using ANCOM-BC, between children with IMD at Day 10 and healthy controls, with volcano plots. Significant genera are plotted according to their log_2_ fold change and adjusted *p*-value. Red dots: IMD at Day 0; blue dots: healthy children. Grey dots mean that their log_2_ fold change was lower than 1, low in magnitude but significant.

**Figure 8:**
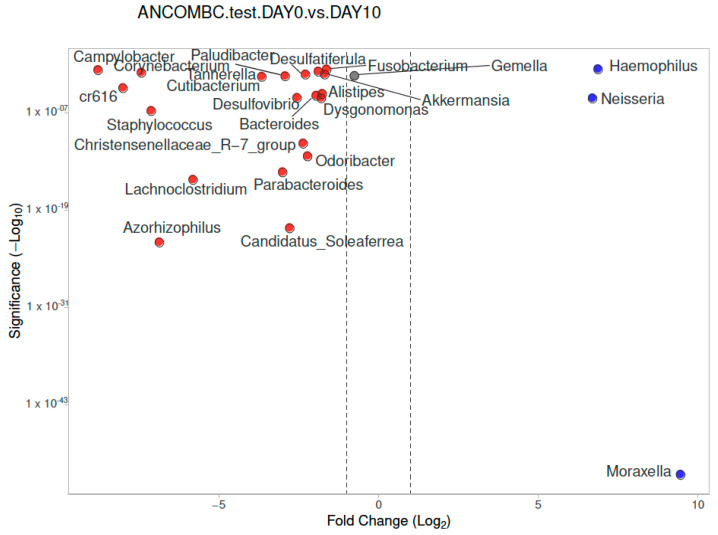
Differential abundance of nasopharyngeal microbiota analysis at genus level, calculated using ANCOM-BC, between children with IMD at Day 0 and Day 10 (paired controls), with volcano plots. Significant genera are plotted according to their log_2_ fold change and adjusted *p*-values. Red dots: IMD at Day 10; blue dots: Day 0.

Differential abundance analysis of genera was carried out using ANCOM-BC for paired comparisons between the samples from the subjects at Day 0 and Day 10, and unpaired comparisons between the children with meningococcemia and the controls (Figure 6, Figure 7 and Figure 8). There was only one difference between the children with IMD at admission and the healthy controls for nasopharyngeal microbiota composition. In the children with IMD at admission, at the genus level, *Neisseria* was significantly more abundant compared to the healthy children (*p* < 0.001; Figure 6). In the children with IMD at Day 10, the *Moraxella* and *Neisseria* genera were reduced compared to in the healthy children, and *Paludibacter*, *Bacteroides, Alistipes, Desulfovibrio, Parabacteroides, Odoribacter*, *and Cutibacterium* were the most abundant genera (Figure 7). In the healthy controls, *Bergeyella, Porphyromonas*, and *Haemophilus* were significantly found to be dominant, compared to the children with IMD at Day 10 (Figure 7). 

In the children with IMD on Day 0, for the paired samples, *Moraxella, Neisseria*, *and Haemophilus* were significantly more abundant compared to the children with IMD at Day 10 (Figure 8). In the children with IMD, at Day 10, the *Moraxella* and *Neisseria* genera were reduced, and 20 different genera were more abundant (shown in Figure 8) compared to Day 0. 

At the species level, *Neisseria meningitidis* serogroup B was shown in six nasopharyngeal samples from the children with IMD at admission (six out of eight IMD cases were caused by serogroup B). At the species level, *Neisseria meningitidis* serogroup B (*p* < 0.05) and *Veillonela parvula* (*p* < 0.001) were significantly more abundant compared to the healthy children. 

## 4. Discussion

In the children with meningococcemia, the intestinal and nasopharyngeal microbiota compositions were found to be different at the time of admission (Day 0) and during follow-up (Day 10) compared to those of the healthy children. This study is the first to demonstrate the importance of the microbiota composition in meningococcemia. 

The risk of certain infections may be associated with specific nasopharyngeal microbiota profiles. The nasopharyngeal microbiota is affected by many factors, mostly genetics, environment, diet, age, and mode of delivery [22,23,24]. Although there is an increasing body of research that demonstrates that the nasopharyngeal microbiota significantly affects susceptibility to respiratory tract infections and the severity of the infection in pediatric patients worldwide [23,25], the relationship between the nasopharyngeal microbiome and meningococcemia has not been demonstrated until now. There has only been one similar study concerning the oropharyngeal microbiome of a college population following a serogroup B meningococcal disease outbreak in Rhode Island, the United States [26]. Retchless and colleagues [26] examined the oropharyngeal microbiota of 158 students, 75 of whom were *N. meningitidis*-positive (the majority were non-groupable, six were serogroup E, and five were serogroup B). They discovered 268 different bacterial taxa, with the most prevalent being *Streptococcus, Veillonella,* and *Rothia* species. Weak relationships between meningococcal carriage and the microbiome makeup were found, as well as risk variables for carriage. The abundance of unidentified *Veillonella species* was adversely linked with *N. meningitidis*. In our study, in the children with IMD at admission, at the genus level, *Neisseria* was significantly more abundant compared to the healthy children. In the children with IMD at Day 10, the *Moraxella* and *Neisseria* genera were reduced, and 20 different genera were more abundant compared to Day 0. In a study examining the relationship between invasive pneumococcal disease (IPD) and nasopharyngeal microbiota, children with IPD exhibited a significantly higher bacterial diversity and richness [27]. However, higher bacterial diversity and richness in children with IPD may also indicate an impaired immune system response. In light of this information, the nasopharyngeal microbiota, specifically, may play a role as a risk or protective factor in the development of invasive disease caused by *N. meningitidis*. The protection of the *Moraxella*-dominant nasopharyngeal microbiota against the need for intensive care has previously been described [28]. However, these studies are related to respiratory tract infection, and it should not be forgotten that, in reality, meningococcemia and sepsis cases in children are based on many factors, such as the interaction between the microbiota and the host characteristics, rather than a single microorganism [27]. 

We first demonstrated certain alterations in the intestinal microbiota of the children with IMD at admission. Regarding the alpha diversity, the Shannon index (a measure of richness and uniformity that considers entropy) was significantly lower in the IMD group compared to the healthy children. There was also a statistical difference in the beta diversity between the stool samples of the children with IMD at admission and the healthy controls. Among the children with IMD at admission, *Enterococcus* were the predominant genera; however, *Faecalibacterium, Lachnospiraceae, Roseburia, Ruminococcus,* and *Blautia* were relatively low in abundance when compared to the healthy controls. Although an imbalance in the homeostasis of the intestinal microbiota is associated with many pathological conditions, the intestinal microbiota and a decrease in commensal species (such as *Faecalibacterium, Blautia,* and *Ruminococcus*) are associated with *Escherichia, Shigella, Salmonella,* and *Enterococcus*, especially in patients with sepsis during follow-up in the pediatric or adult intensive care units [29]. *Faecalibacterium spp., Blautia spp.,* and *Ruminococcaceae spp.* are known to produce small-chain fatty acids, which have been shown to be significantly lower in critically ill patients compared to healthy patients [9]. Our findings also show decreased *Faecalibacterium* in the IMD group compared to the healthy control group. Decreased *F. prausnitzii* has been reported in certain chronic disorders, inflammatory bowel disease, and metabolic disorders [30]. We previously also found that *F. prausnitzii* was decreased in the intestinal microbiota composition of cases of multi-inflammatory syndrome in children (MIS-C) [31]. The course of acute IMD is rapid, and clinical findings have been observed within hours. Mortality has been observed within the first 24 h of the disease, despite therapeutic interventions. In our study, all IMD cases were previously healthy, and the fecal and nasopharyngeal samples were collected within the first hours of admission. For this reason, the microbiota composition changes were related to the infection itself and/or the predisposition to severe infections. Alterations in the intestinal microbiota composition, called dysbiosis, might play a role in the infection and/or severity.

We also found certain microbiota composition changes at Day 10 in the IMD group compared to the healthy controls. At Day 10, in the children with IMD, *Enterococcus, Pseudomonas, Granulicatella, Prevotella, Eggerthella, Actinomyces, Lactobacillus,* and *Streptococcus* were found to be significantly dominant, while in the healthy controls, *Lachnospiraceae NK4A136 group, Roseburia, Ruminococcaceae UCG-013, Agathobacter, Ruminococcus_1,* and more than 20 different genera were significantly found to be dominant compared to the children with IMD at Day 10. Intestinal microbiota composition changes during the intensive care unit stay were due to different factors such as medication, including drugs, nutrition, and others. Dysbiosis may make patients more susceptible to multiorgan failure syndrome and hospital-acquired infections [32]. Building a healthy intestinal microbiota may be important for improving outcomes in critically ill patients, as the gut is assumed to play a central role in the progression to critical illness, sepsis, and multi-organ failure [33].

Our study has some limitations. We included ten children with IMD. The sample size was small; however, meningococcemia is relatively rare compared to other pediatric infections, and we attempted to standardize our cohort regarding age, sampling time, and the inclusion and exclusion criteria. While IMD is also common in children under three years old, we did not include this age group because the microbiota composition is not stable in the first 2–3 years of life, and there are defined risk factors affecting the microbiota composition in the first 1000 days of life. Our study is independent of the limitations of the dysbiosis effect due to antibiotic use before the admission of the patients. We had no opportunity to evaluate the effect of previous nutritional preferences and lifestyle before admission and enteral nutrition or other dietary interventions for the intestinal microbiota composition during the patients’ intensive care unit stays.

Until now, there has been no study in which the intestinal and nasopharyngeal microbiota were evaluated by next-generation sequencing analyses in children with IMD. We first found alterations in the intestinal and nasopharyngeal microbiota composition in the children with IMD. The disruption of the microbiota’s integrity can enhance vulnerability to serious infections because of the beneficial effects of a healthy microbiota on colonization resistance and systemic immunity [9]. In this context, understanding the interaction of microbiota with pathogens, e.g., *N. meningitidis*, could give us the opportunity to understand the disease’s dynamics.

## Figures and Tables

**Table 1:** Fecal and nasopharyngeal alpha diversity indices (Shannon index and Chao-1 index) comparison between meningococcemia group and healthy controls.

	Meningococcemia		Healthy Controls
	Day 0	Day 10	
**Fecal**			
Shannon index	4.27 ± 1.40 ^a^	3.10 ± 0.87 ^b^	5.60 ± 0.42
Chao1 index	161 ± 74	100 ± 53 ^c^	215 ± 51
**Nasopharyngeal**			
Shannon index	2.43 ± 0.76 ^d^	5.29 ± 0.94	4.14 ± 2.25
Chao1 index	122 ± 42 ^e^	230 ± 735	186 ± 79

^a^ Fecal, meningococcemia group vs. control *p* = 0.002, Day 0; ^b^ Fecal, meningococcemia group vs. control *p* = 0.001, Day 10; ^c^ Fecal, meningococcemia group vs. control *p* = 0.001, Day 10; ^d^ nasopharyngeal, meningococcemia group, Day 0 vs. meningococcemia group, Day 10; *p* = 0.001; ^e^ nasopharyngeal, meningococcemia group, Day 0 vs. meningococcemia group, Day 10; *p* = 0.002.

## Data Availability

The raw amplicons data of the INMACS-PICU Study have been deposited in the European Nucleotide Archive (ENA) under project number PRJEB59932 with accession numbers ERS14646173-ERS14646221.

## Supplementary Materials

The following supporting information can be downloaded at: https://www.mdpi.com/article/10.3390/diagnostics13121984/s1, Figure S1. The flow chart is shown according to Strengthening the Organization and Reporting of Microbiome Studies (STORMS).

## References

[1] Acevedo R., Bai X., Borrow R., Caugant D.A., Carlos J., Ceyhan M., Christensen H., Climent Y., De Wals P., Dinleyici E.C. (2019). The Global Meningococcal Initiative meeting on prevention of meningococcal disease worldwide: Epidemiology, surveillance, hypervirulent strains, antibiotic resistance and high-risk populations. Expert Rev. Vaccines.

[2] Alderson M.R., Arkwright P.D., Bai X., Black S., Borrow R., Caugant D.A., Dinleyici E.C., Harrison L.H., Lucidarme J., McNamara L.A. (2022). Surveillance and control of meningococcal disease in the COVID-19 era: A Global Meningococcal Initiative review. J. Infect..

[3] Ceyhan M., Ozsurekci Y., Basaranoglu S.T., Gurler N., Sali E., Emiroglu M.K., Oz F.N., Belet N., Duman M., Ulusoy E. (2020). Multicenter Hospital-Based Prospective Surveillance Study of Bacterial Agents Causing Meningitis and Seroprevalence of Different Serogroups of Neisseria meningitidis, Haemophilus influenzae Type b, and Streptococcus pneumoniae during 2015 to 2018 in Turkey. Msphere.

[4] Tekin R.T., Dinleyici E.C., Ceyhan M., Karbuz A., Salman N., Sutçu M., Kurugol Z., Balliel Y., Celik M., Hacimustafaoglu M. (2017). The prevalence, serogroup distribution and risk factors of meningococcal carriage in adolescents and young adults in Turkey. Hum. Vaccines Immunother..

[5] Kizil M.C., Kilic O., Ceyhan M., Nepesov M.I., Karbuz A., Kurugol Z., Hacimustafaoglu M., Celebi S., Dinleyici M., Carman K.B. (2021). Nasopharyngeal Meningococcal Carriage among Children and Adolescents in Turkey in 2018: An Unexpected High Serogroup X Carriage. Children.

[6] Hou K., Wu Z.-X., Chen X.-Y., Wang J.-Q., Zhang D., Xiao C., Zhu D., Koya J.B., Wei L., Li J. (2022). Microbiota in health and diseases. Signal Transduct. Target. Ther..

[7] Libertucci J., Young V.B. (2018). The role of the microbiota in infectious diseases. Nat. Microbiol..

[8] Wu H.J., Wu E. (2012). The role of gut microbiota in immune homeostasis and autoimmunity. Gut Microbes.

[9] Haak B.W., Wiersinga W.J. (2017). The role of the gut microbiota in sepsis. Lancet Gastroenterol. Hepatol..

[10] Miller W.D., Keskey R., Alverdy J.C. (2020). Sepsis and the Microbiome: A Vicious Cycle. J. Infect. Dis..

[11] Wozniak H., Beckmann T.S., Fröhlich L., Soccorsi T., Le Terrier C., de Watteville A., Schrenzel J., Heidegger C.-P. (2022). The central and biodynamic role of gut microbiota in critically ill patients. Crit. Care.

[12] Kean I.R.L., Wagner J., Wijeyesekera A., De Goffau M., Thurston S., Clark J.A., White D.K., Ridout J., Agrawal S., Kayani R. (2022). Profiling gut microbiota and bile acid metabolism in critically ill children. Sci. Rep..

[13] Dickson R.P., Singer B.H., Newstead M.W., Falkowski N.R., Erb-Downward J.R., Standiford T.J., Huffnagle G.B. (2016). Enrichment of the lung microbiome with gut bacteria in sepsis and the acute respiratory distress syndrome. Nat. Microbiol..

[14] Wiertsema S.P., van Bergenhenegouwen J., Garssen J., Knippels L.M.J. (2021). The Interplay between the Gut Microbiome and the Immune System in the Context of Infectious Diseases throughout Life and the Role of Nutrition in Optimizing Treatment Strategies. Nutrients.

[15] Callahan B.J., Mcmurdie P.J., Rosen M.J., Han A.W., Johnson A.J.A., Holmes S.P. (2016). DADA_2_: High-resolution sample inference from Illumina amplicon data. Nat. Methods.

[16] Langmead B., Salzberg S.L. (2012). Fast gapped-read alignment with Bowtie 2. Nat. Methods.

[17] Caporaso J.G., Kuczynski J., Stombaugh J., Bittinger K., Bushman F.D., Costello E.K., Fierer N., Gonzalez Peña A., Goodrich J.K., Gordon J.I. (2010). QIIME allows analysis of high-throughput community sequencing data. Nat. Methods.

[18] R Development Core Team (2013). A Language and Environment for Statistical Computing.

[19] Lin H., Das Peddada S. (2020). Analysis of compositions of microbiomes with bias correction. Nat. Commun..

[20] Goedhart J., Luijsterburg M.S. (2020). VolcaNoseR is a web app for creating, exploring, labeling and sharing volcano plots. Sci. Rep..

[21] Mirzayi C., Renson A., Furlanello C., Sansone S.-A., Zohra F., Elsafoury S., Geistlinger L., Kasselman L.J., Eckenrode K., van de Wijgert J. (2021). Reporting guidelines for human microbiome research: The STORMS checklist. Nat. Med..

[22] Dubourg G., Edouard S., Raoult D. (2019). Relationship between nasopharyngeal microbiota and patient’s susceptibility to viral infection. Expert Rev. Anti-Infect. Ther..

[23] Esposito S., Principi N. (2017). Impact of nasopharyngeal microbiota on the development of respiratory tract diseases. Eur. J. Clin. Microbiol. Infect. Dis..

[24] Schenck L.P., Surette M.G., Bowdish D.M.E. (2016). Composition and immunological significance of the upper respiratory tract microbiota. FEBS Lett..

[25] Flynn M., Dooley J. (2021). The microbiome of the nasopharynx. J. Med. Microbiol..

[26] Retchless A.C., Kretz C.B., Rodriguez-Rivera L.D., Chen A., Soeters H.M., Whaley M.J., Wang X. (2020). Oropharyngeal microbiome of a college population following a meningococcal disease outbreak. Sci. Rep..

[27] Camelo-Castillo A., Henares D., Brotons P., Galiana A., Rodríguez J.C., Mira A., Muñoz-Almagro C. (2019). on behalf of the Catalan Study Group of Host- Pathogen Interaction in Patients With IPD. Nasopharyngeal Microbiota in Children with Invasive Pneumococcal Disease: Identification of Bacteria With Potential Disease-Promoting and Protective Effects. Front. Microbiol..

[28] Luna P.N., Hasegawa K., Ajami N.J., Espinola J.A., Henke D.M., Petrosino J.F., Piedra P.A., Sullivan A.F., Jr. C.A.C., Shaw C.A. (2018). The association between anterior nares and nasopharyngeal microbiota in infants hospitalized for bronchiolitis. Microbiome.

[29] Moron R., Galvez J., Colmenero M., Anderson P., Cabeza J., Rodriguez-Cabezas M.E. (2019). The Importance of the Microbiome in Critically Ill Patients: Role of Nutrition. Nutrients.

[30] Ferreira-Halder C.V., de Sousa Faria A.V., Andrade S.S. (2017). Action and function of Faecalibacterium prausnitzii in health and disease. Best Pract. Res. Clin. Gastroenterol..

[31] Suskun C., Kilic O., Ciftdogan D.Y., Guven S., Karbuz A., Parlakay A.O., Kara Y., Kacmaz E., Sahin A., Boga A. (2022). Intestinal microbiota composition of children with infection with severe acute respiratory syndrome coronavirus 2 (SARS-CoV-2) and multisystem inflammatory syndrome (MIS-C). Eur. J. Pediatr..

[32] Liu Z., Li N., Fang H., Chen X., Guo Y., Gong S., Niu M., Zhou H., Jiang Y., Chang P. (2019). Enteric dysbiosis is associated with sepsis in patients. FASEB J..

[33] Agudelo-Ochoa G.M., Valdés-Duque B.E., Giraldo-Giraldo N.A., Jaillier-Ramírez A.M., Giraldo-Villa A., Acevedo-Castaño I., Yepes-Molina M.A., Barbosa-Barbosa J., Benítez-Paéz A. (2020). Gut microbiota profiles in critically ill patients, potential biomarkers and risk variables for sepsis. Gut Microbes.

