# Supplementary material for: Analysis of Intestinal and Nasopharyngeal Microbiota of Children with Meningococcemia in Pediatric Intensive Care Unit: INMACS-PICU Study
Source: Diagnostics (Basel). 2023 Jun 6;13(12):1984. doi: 10.3390/diagnostics13121984 (PMC10296867; doi:10.3390/diagnostics13121984)

Supplementary Figure S1. The flow chart is shown according to Strengthening the Organization and Reporting of Microbiome Studies (STORMS) (Mirzayi C et al. Nature Medicine 2021) [21].

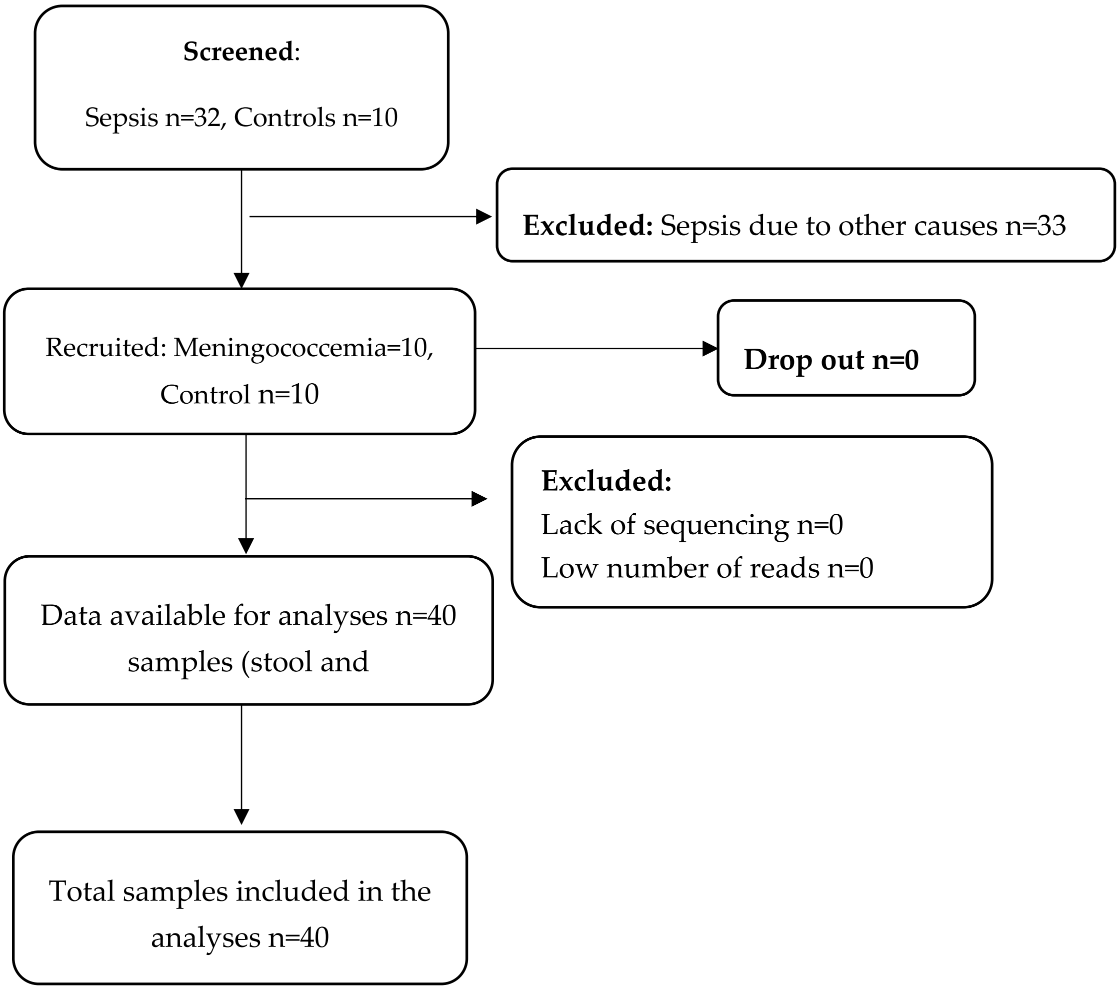

Supplement: Supplementary file 1 [file diagnostics-13-01984-s001.zip › diagnostics-2269653-supplementary.pdf]
